# Upper First and Second Molar Pulp Chamber Endodontic Anatomy Evaluation According to a Recent Classification: A Cone Beam Computed Tomography Study

**DOI:** 10.3390/jimaging10010009

**Published:** 2023-12-28

**Authors:** Rodolfo Reda, Dario Di Nardo, Alessio Zanza, Valentina Bellanova, Rosemary Abbagnale, Francesco Pagnoni, Maurilio D’Angelo, Ajinkya M. Pawar, Massimo Galli, Luca Testarelli

**Affiliations:** 1 Department of Oral and Maxillo Facial Sciences, Sapienza University of Rome, Via Caserta 6, 00161 Rome, Italy; rodolfo.reda@uniroma1.it (R.R.); valentinabellanova98@gmail.com (V.B.); rose.abbagnale@gmail.com (R.A.); francesco.pagnoni22@gmail.com (F.P.); maurilio.dangelo@uniroma1.it (M.D.); luca.testarelli@uniroma1.it (L.T.); 2 Operative Research Unit of Dentistry, Policlinico Universitario Campus Bio-Medico Foundation, Via Alvaro del Portillo 200, 00128 Rome, Italy; dario.dinardo@uniroma1.it; 3 Department of Conservative Dentistry and Endodontics, Nair Hospital Dental College, Mumbai 400008, India

**Keywords:** upper first molar, upper second molar, CBCT, pulp floor, classification

## Abstract

(1) The possibility of knowing information about the anatomy in advance, in particular the arrangement of the endodontic system, is crucial for successful treatment and for avoiding complications during endodontic therapy; the aim was to find a correlation between a minimally invasive and less stressful endodontic access on Ni-Ti rotary instruments, but which allows correct vision and identification of anatomical reference points, simplifying the typologies based on the shape of the pulp chamber in coronal three-dimensional exam views. (2) Based on the inclusion criteria, 104 maxillary molars (52 maxillary first molars and 52 maxillary second molars) were included in the study after 26 Cone Beam Computed Tomography (CBCT) acquisitions (from 15 males and 11 females). And linear measurements were taken with the CBCT-dedicated software for subsequent analysis. (3) The results of the present study show data similar to those already published about this topic. Pawar and Singh’s simplified classification actually seems to offer a schematic way of classification that includes almost all of the cases that have been analyzed. (4) The use of a diagnostic examination with a wide Field of View (FOV) and low radiation dose represents an exam capable of obtaining a lot of clinical information for endodontic treatment. Nevertheless, the endodontic anatomy of the upper second molar represents a major challenge for the clinician due to its complexity both in canal shape and in ramification.

## 1. Introduction

The possibility of knowing the canal anatomy in advance offers the clinician the possibility of making the treatment safer, reducing the possibility of stressing the instruments and speeding up the treatment [1,2,3]. Although the two-dimensional periapical digital radiography represents, in the diagnostic and therapeutic protocol, the main diagnostic exam prescribed, its limitations are described below [4]. 

This is especially true with reference to the specific analysis of the pulp chamber floor [5].

Furthermore, the internal and external morphological configurations of roots and root canals, especially of multi-rooted teeth, are quite complex [6,7]. 

The possibility of knowing this information in advance, in particular the arrangement of the root canals, is crucial for successful treatment and for avoiding complications during endodontic therapy [4,8]. 

The risk of performing an endodontic treatment without having understood the dental anatomy, a fundamental factor for carrying out a minimally invasive and low-risk endodontic treatment, represents a condition of extreme danger of torsional and flexural overload of the endodontic instruments, which can lead to their intracanal separation [9,10,11]. 

To address such clinical circumstances, Pawar and Singh proposed a new classification in 2020, labeled “Pawar and Singh Molar Pulp Chamber Floor Classification”. In this classification, the shape of the pulp chamber floor is described based on the location and number of root canal orifices present, resulting in a unique alphabetic letter “K, Y, I” categorization [12,13,14]. 

Knowledge of the positioning of the canal orifices, the mutual distance and the volumes of the pulp chamber allows us to know the areas of less dentin thickness to be respected with less aggressive canal instruments to avoid the risk of fracture of the endodontically treated tooth [15,16]. In this way, it is possible to reduce the volume of the chamber opening necessary for endodontic treatment, although this does not substantially influence the resistance of the tooth in the long term [16,17]. 

In light of the inclusion of the upper first molars and upper second molars in this diagnostic examination, the anatomical characteristics of the chamber floor and their endodontic anatomy were analyzed with a low-dose and wide image field Cone Beam Computed Tomography (CBCT), now able to offer great image reading quality, even of soft tissues [18,19].

Considering the recent improvement in diagnostic imaging methods, it is increasingly common to have high-resolution exams capable of orienting the diagnosis and directing towards the most correct therapeutic choice and, in the case of endodontic treatment, the choice of the most suitable rotary instruments, thereby reducing the risk of intra-canal separation by allowing direct access to the canal. 

Furthermore, it is interesting to remember that the availability of an examination of this type allows us to acquire all the indications to proceed even with a surgical endodontic treatment, analyzing the relationships with the neighboring noble structures or the depth of the root apex to calibrate the depth of the bone osteotomy [20,21]. 

The aim of this study is to analyze the anatomy of the pulp chamber in relation to the endodontic anatomy of the upper first and second molars applying a recently proposed classification. The objective was to find a correlation between a minimally invasive and less stressful endodontic access on Ni-Ti rotary instruments, but which allows correct vision and identification of anatomical reference points, simplifying the typologies based on the shape of the pulp chamber in coronal 3D exam views.

## 2. Materials and Methods

This retrospective study was conducted in accordance with the Declaration of Helsinki. Each evaluation was conducted using linear measurements enabled by the CBCT software Sidexis Galileos Implant, v. 9.1 (Dentsply Sirona, Wals bei Salzburg, Austria). 

All CBCT scans analyzed had been previously acquired for reasons other than the present research, prescribed for planning oral surgery, third molar extractions and implant placement in the upper jaw. 

CBCT scans were performed with the Orthophos SL 3D imaging unit (Dentisply Sirona, Wals bei Salzburg, Austria) set at 500 ms, 7 mA, 85 kV, FOV of 8 cm × 8 cm and resolution of 0.16 mm; the software used for image analysis was Sidexis Galileos Implant, v. 9.1 (Dentsply Sirona). The inclusion criteria were developed taking into consideration already published manuscripts on this topic.

The inclusion criteria to include maxillary first and second molars were as follows [5]:

- the presence of four maxillary molars (including jus first and second);
- no previous conservative or endodontic treatment;
- absence of coronal restorations;
- complete root formation;
- absence of root canal calcification—root canals should be visible from the pulp chamber to the apical part;
- absence of conditions that could limit the possibility of identifying the structures being studied, such as neoplasms, cysts, large periradicular lesions, artifacts, or internal or external root resorptions;
- no influence of the wisdom tooth on the shape of the roots and canals of the seventh due to the close position;
- older than 18;
- patients of Caucasian origin.

Based on the inclusion criteria, 104 maxillary molars (52 maxillary first molars and 52 maxillary second molars) were included in the study after 26 CBCT acquisitions (from 15 males and 11 females) [5,7].

In order to obtain the standardization of the measures, first, each tooth analyzed was realigned according to its long axis [5,12]. To clinically identify the long axis of the tooth, the FACC (Facial Axis of the Clinical Crown) was used, drawing on the center of the prominence of the central lobe on the vestibular surface of all teeth, except for the molars, in which it was located along the sulcus dividing the buccal cusps.

To schematically indicate the teeth under study, the FDI notation was used, which uses a two-digit numbering system in which the first digit represents a tooth’s quadrant and the second digit represents the number of the tooth from the midline of the face. To indicate the upper first molars, the formula 16,26-M1 will be used; for the upper second molars, 17,27-M2 will be used.

From a radiographic point of view, it was evaluated through the orientation of a straight line, in the sagittal section, between the occlusal plane and the apex of the palatal root [5]. 

Subsequently, evaluations were carried out at the level of the CEJ (Cement–Enamel Junction), 5 mm apical from the CEJ and 3 mm coronal from the apex. 

At these levels, the following characteristics were studied [5,7,12]:

- long and short diameter of the pulp chamber floor;
- shape of the pulp chamber associated with a letter of the alphabet (in relation to the number and arrangement of canal orifices);
- diameter of root canal orifices;
- distance between intra- and inter-root canals;
- possible fusion of the roots;
- root diameter.

All of these factors are responsible for predicting the anatomy of the pulp chamber floor [5,10,12].

The classification of maxillary molars includes the description of the shape of the floor of the pulp chamber, mainly divided into three classes: K, Y and I [12,14]. 

Depending on the position and number of canal orifices that form a particular letter of the alphabet, the classification is the following:

1. K: in maxillary molars with the presence of four canal orifices: MB, MB-2, disto-buccal and palatal; a line joining the disto-buccal and palatal and two other lines radiating from the center of this line to MB and MB-2, forming the letter “K” of the alphabet.
2. Y: in maxillary molars with three canal orifices: MB, disto-buccal and palatal; when a line is formed joining the three canals in the center of the access cavity, it resembles the letter “Y” of the alphabet.
3. I: in maxillary molars with two canal orifices: buccal and palatal; a line joining both canals, resembling the letter “I” of the alphabet [5,12,14].

All measurements were performed by two calibrated operators (V.B. and R.R.) after having performed the analysis of the first 5 CBCTs together. The level of agreement on measurement consistency was assessed using the kappa statistic.

Statistical data analysis was performed using dedicated software (IBM Corp. Released 2013. IBM SPSS Statics for Windows, version 22.0. Armonk, NY, USA: IBM Corp).

Descriptive statistics are provided by presenting means and standard deviations, data and frequencies for categorical variables. For normally distributed continuous variables, differences between groups were calculated using Student’s *t*-test (one-sample *t*-test and two-sample paired *t*-test). The level of significance was set to alpha = 0.05 and the significance value was *p*-value ≤ 0.05.

## 3. Results

The results of the present study show data similar to those already published about this topic. Pawar and Singh’s simplified classification seems to offer a schematic way of classification that includes almost all of the cases that have been analyzed. 

All the characteristics analyzed are summarized in Table 1. 

The 17,27-M2 are smaller (based on the assessments of the long and short diameter of each tooth) and have a pulp chamber with an anatomy that is very often simplified. 

Through this examination, the “Y” conformations were found to be extremely frequent for 17,27-M2 (in 21 17,27-M2 compared to 6 16,26-M1), which, however, show an extremely intricate endodontic system despite the greater simplicity shown in the analysis of the pulp chamber floor. 

**Figure 1:**
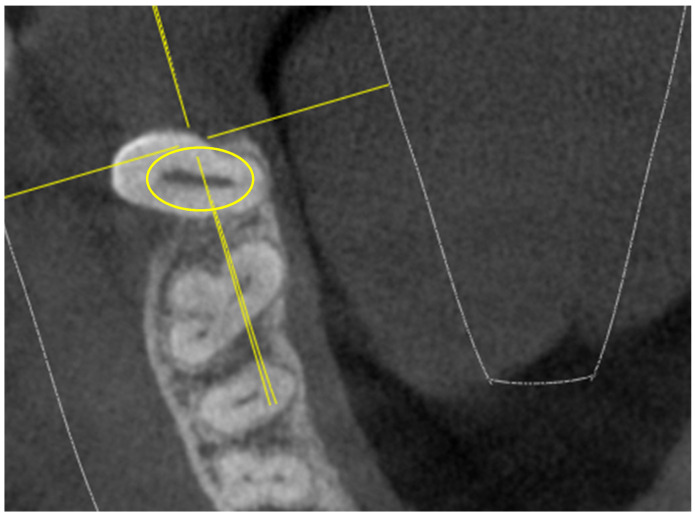
Tooth 17 with “I” pulp chamber floor (in the yellow ring).

The “I” conformation was detected in rare cases, specifically in 3 16,26-M1 and in 4 17,27-M2 (out of 52 16,26-M1 and 52 17,27-M2), as indicated in Figure 1.

The diameter values of the mesio-buccal and disto-buccal canal orifices are similar; the palatals appear to be larger and the MB2s have smaller diameters (0.3–0.4 mm), as already known in the literature.

In 17,27-M2, the presence of MB2s, at several levels of the mesial root, is close to the data presented in the literature, bordering on 65%. 

**Figure 2:**
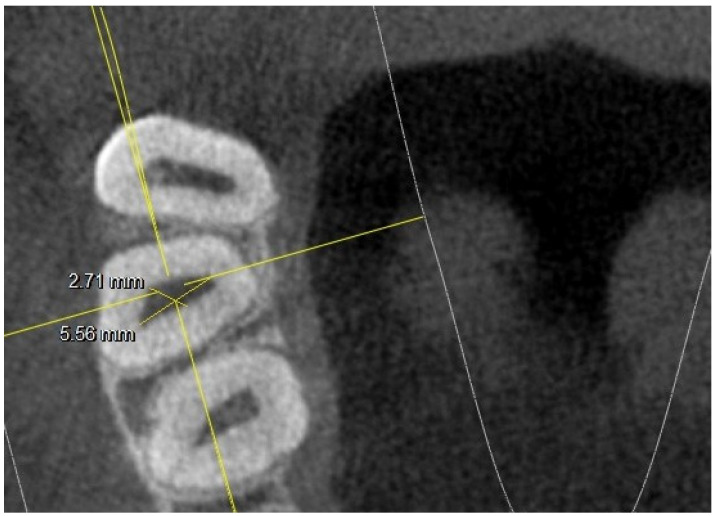
Tooth 17 with 4 canals at chamber floor level; major and minor axis measurements highlighted.

There were no statistically significant differences (*p* difference 0.078) between the right side and the left side in the observed population, although the chamber conformation was sometimes different. In several 17,27-M2, the MB2 canal was radiographically found at the level of the chamber floor, as shown in Figure 2.

This figure also shows an example of how the measurements at the level of the chamber floor were conducted (diameter along and short diameter).

**Figure 3:**
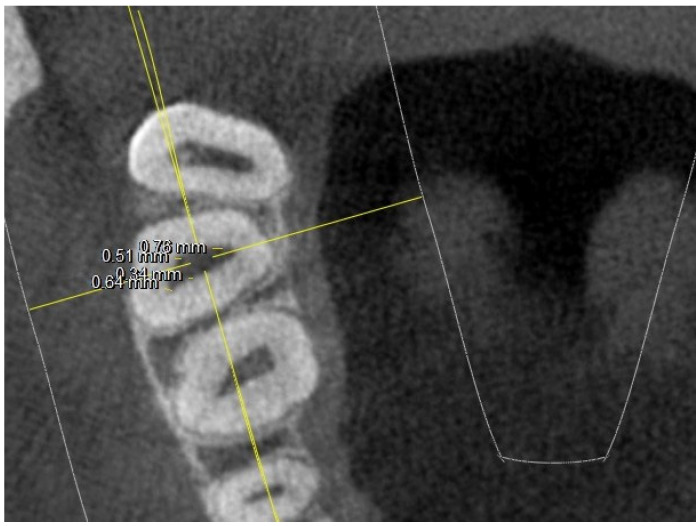
Root canal measurements at pulp chamber floor level.

Where four canals were found at the chamber floor level, linear measurements of the canal diameter were conducted as shown in Figure 3.

At the pulp chamber floor level, in some teeth, especially the 17,27-M2, it was not possible to detect the entrance to the MB2 canal, although it was visible in subsequent scans in the apical direction. In the upper first molars, in 9 cases out of 52, the MB2 was not evident at the level of the CEJ, while at 5 mm from the CEJ, the effective absence of the MB2 was relative only to five. 

From this, it can be deduced that in 4 teeth the orifice of the MB2 was located more apically than the openings of the other canals; instead, as regards the 17,27-M2, in 25 cases out of 52, the MB2 was not evident at the level of the CEJ, while at 5 mm from the CEJ, the effective absence of the MB2 was relative only to 18 teeth. It can therefore be concluded that in seven teeth the orifice of the MB2 was located more apically than the other orifices. 

The distance of the MB2 from the mesial buccal is about 1.5–1.6 mm; instead, the distance between the MB2 and the palatal is around 3.5–3.6 mm.

As far as the 16,26-M1 are concerned, a total of 47 MB2s were detected at 5 mm apical from the CEJ; however, at 3 mm coronal to the apex, this number is reduced to 36. 

It is possible to justify this result by considering the possible confluence of the MB2 in the MV in about 25% of cases or because they may be completely independent.

In the 17,27-M2, however, 34 MB2s were detected at 5 mm from the CEJ; however, at 3 mm from the apex, this number is reduced to 18. This is justified by the possible confluence of the MB2 in the MV in just under 50% of cases.

**Figure 4:**
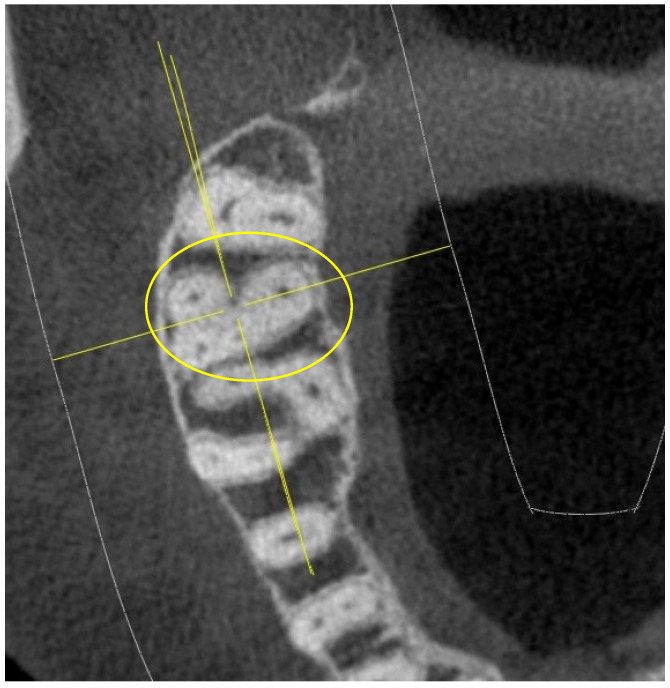
Root canal sections 5 mm apical from the CEJ on a 17.

In the following sections, the finding of the MB2 canal was much more frequent, as shown in Figure 4.

**Figure 5:**
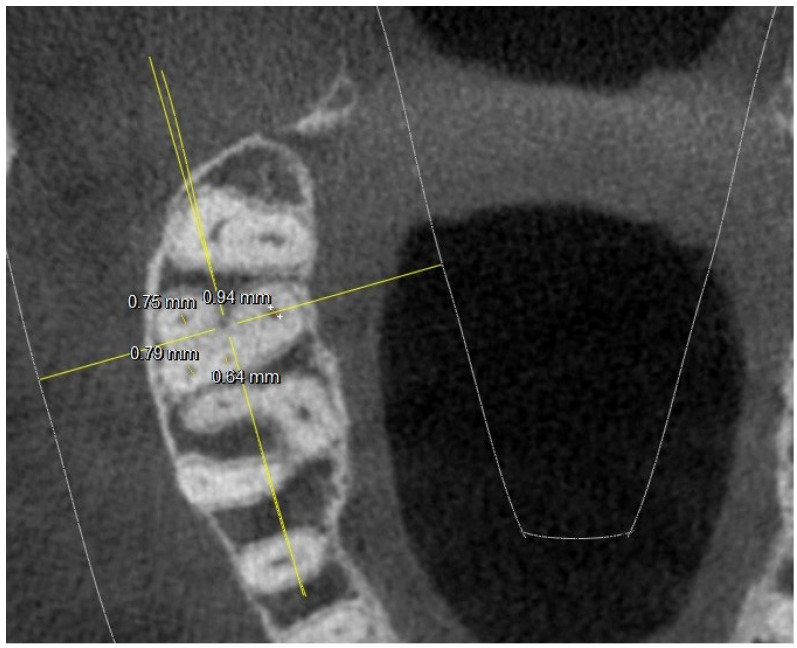
Measurements of the root canal in a section 5 mm apical from the CEJ on a 17.

In these sections, the diameters of the canal lumen in these canals were measured linearly, as shown in Figure 5.

**Figure 6:**
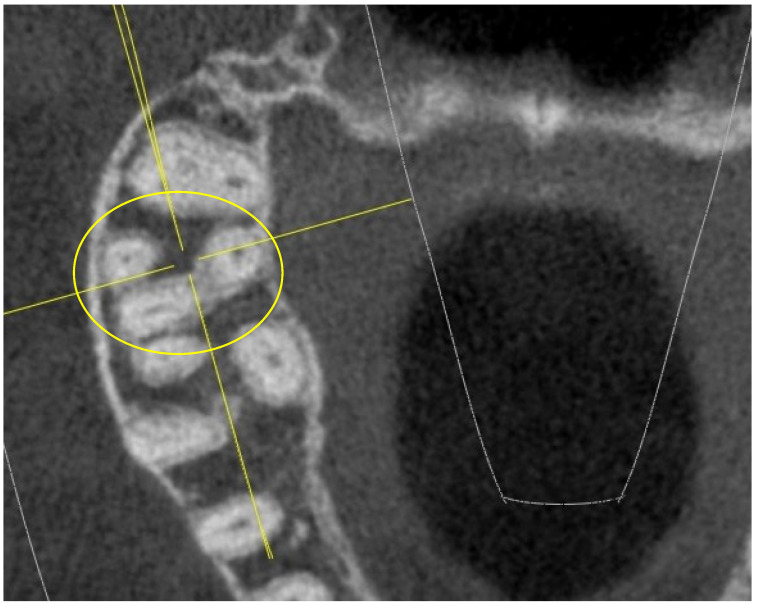
Root canal diameters of the sections 3 mm coronal from the apex, 17.

The subsequent radiographic analysis was conducted 3 mm from the apex, to evaluate the complexity of the endodontic system by comparing it with the section 5 mm apical to the CEJ, as shown in Figure 6.

**Figure 7:**
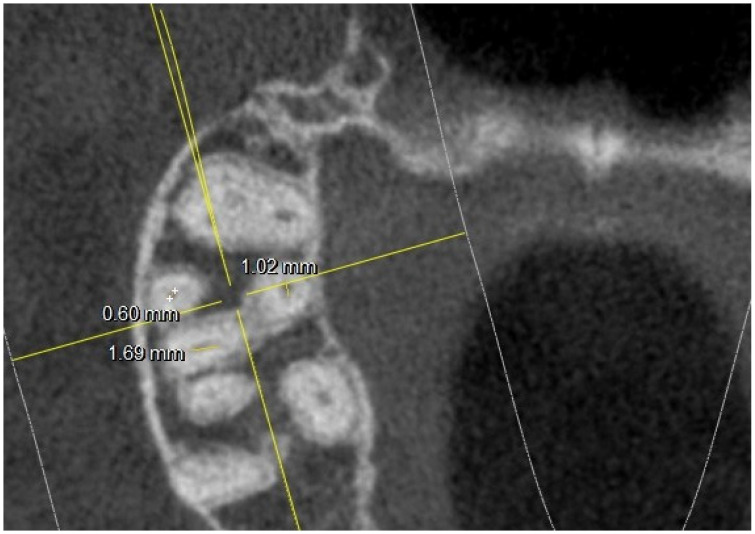
Root canal diameter measurements 3 mm coronal to the apex on a 17.

In the subsequent analysis, the diameters of the canal lumen were measured at 3 mm coronal to the apex, as shown in Figure 7.

**Figure 8:**
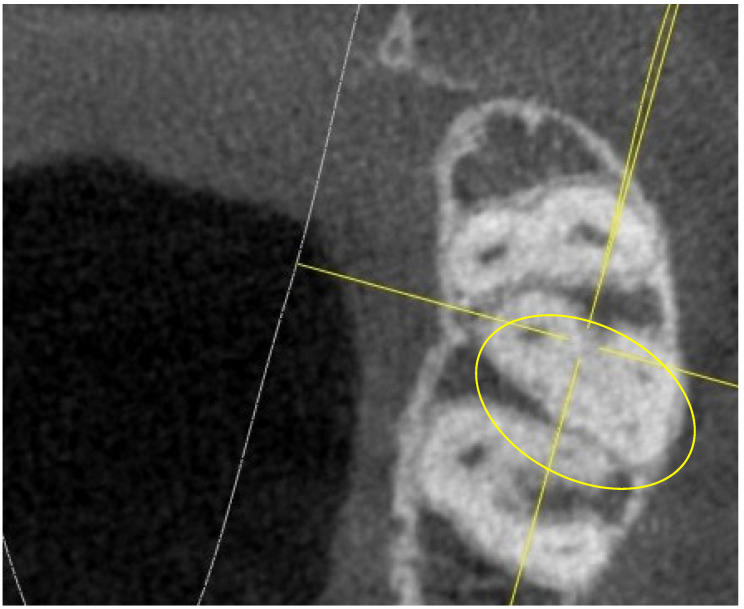
Tooth 27 section 5 mm apical to the CEJ showing 4 canals.

It is interesting to note that the number of upper sections which already showed a complex chamber anatomy 5 mm from the CEJ, similar to that of the upper sections, was really high (34 upper sections with four canals out of 52 total), as shown in Figure 8.

**Figure 9:**
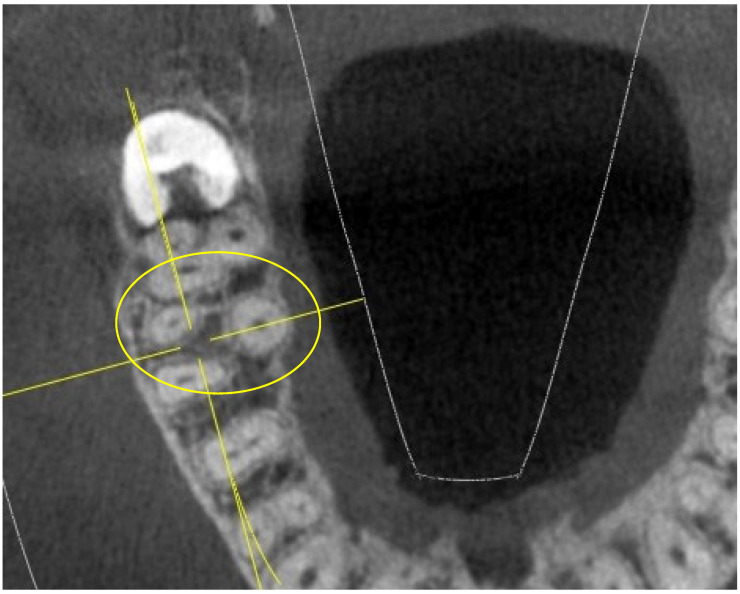
Section 5 mm apical from the CEJ showing 16 with a ribbon-shaped mesial canal.

In the 16,26-M1, the presence of four canals was detected in 47 teeth out of a total of 52. It is necessary to consider how the 16,26-M1, although in a considerably lesser way than the 17,27-M2, (6 16,26-M1 out of 52 total) also showed variable anatomies at the chamber level or at 5 mm from the CEJ, as shown in Figure 9.

**Figure 10:**
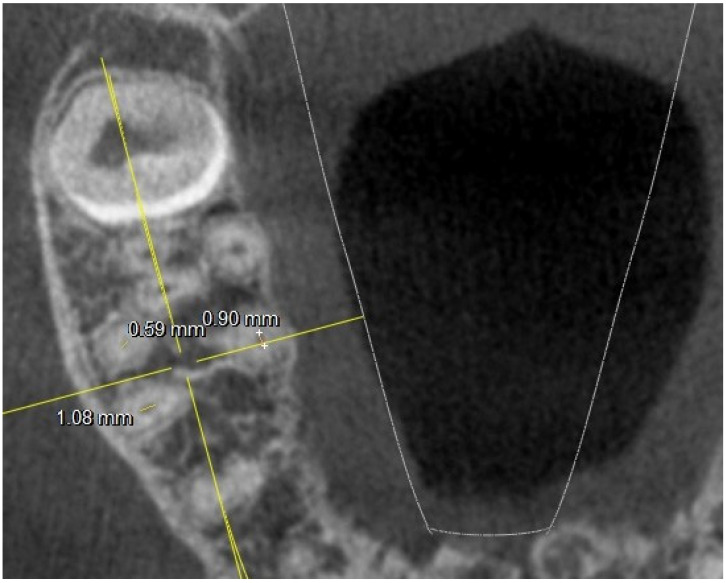
Section 5 mm from the CEJ measurement showing a 16 with a ribbon-like mesial canal.

Even in this case, the diameters of the canals at different heights were measured, emphasizing the cases in which the canal was not circular or oval in shape but had different sections, such as this case in which it is ribbon-like (Figure 10).

In the 17,27-M2 instead, the presence of ribbon-like canals was found in a greater number (14 total).

The percentage of fused roots in the middle third and in the apical third is greater in the 17,27-M2 than in the first molars with a double frequency. The 16,26-M1 usually have diverging roots; the 17,27-M2 on the other hand, often, also considering the proximity to the 18,28-M3, have a root anatomy converging in an apical direction.

## 4. Discussion

The preliminary evaluation of the endodontic anatomy is a fundamental aspect in the simplification of a clinical procedure often considered complex due to the reduced visibility and difficulty of accessing the endodontic system [4,5]. 

This system often has complex and branched anatomies with branches that only chemical cleansing is able to reach and disinfect [22,23]. 

Furthermore, the complexity of the endodontic system, if unexpected, can represent a criticality in terms of cumulative stress on the Ni-Ti rotary instruments in their use in unexpectedly complex canals [9,24].

Considering the results obtained, the root anatomy of the 17,27-M2 and 16,26-M1 represents an ever-present challenge for correct endodontic therapy (as indicated in Table 1). 

The disinfection of the root canal system, with its many ramifications, represents one of the fundamental points of the therapy for obtaining success [22]. 

This condition is favored by correct pulp chamber access and by the adequate instrumentation of the floor of the pulp chamber itself to favor a linear entry of the endodontic rotary instruments, a condition capable of greatly reducing the stresses to which the instruments are subjected in their intracanal rotation [24]. 

The ratios between the dimensions of the 16,26-M1 agree with the already published data [25,26]. From the analysis of the chamber floor at the height of the other orifices, it is possible to obtain information on the presence of MB2s in most of the teeth analyzed (47 16,26-M1 with MB2 out of 52 total and 34 17,27-M2 with MB2 out of 52 total), with a symmetry right/left that is particularly constant and with differences that are never statistically significant, unless we are dealing with chamber floors with an “I” shape in the 17,27-M2, which are an occasional find (4 17,27-M2 with an “I” shape out of a total of 52) and never symmetrical. 

In an extremely small percentage, the 17,27-M2 showed an extremely complex “I”-shaped chamber floor conformation, which particularly increases the risk of perforations or false paths during endodontic practice (Figure 1).

The “K” conformations, which appear with a relative frequency of 27 out of 52, are about half compared to the same chamber anatomical scheme at the level of the 16,26-M1. The detection of MB2 at the chamber floor level, therefore, occurred in 65% of cases.

The Y-shaped anatomy is present in 6 cases at the level of the 16,26-M1 and in 21 cases for the 17,27-M2. But the effective number of MB2s at 5 mm from the CEJ was higher than that detected at the same level. This reinforces the concept, which has been clear for some time now, of searching for the MB2 canal at a slightly more apical level than the rest of the canal system in its coronal portion, removing part of the dentin tissue that is often found above this orifice, hiding its entrance [25,26]. 

Furthermore, it has been reported that, in some of these cases, an accessory buccal canal system was not present. As briefly described in the results, the accessory buccal system, in many of the 17,27-M2 analyzed, was manifested in the radiographic section, but only in the apical portions with respect to the reference chamber one, in which the other canal openings were evident. This strengthens the clinical evidence that in many cases the MB2 canal is located apically compared to the rest of the root canal system. This operation often forces the clinician to proceed with the removal of dentin tissue, which obliterates the lumen of the MB2 canal, increasing the risk of chamber perforation or false roads.

In the literature, the presence of the MB2 canal in the 16,26-M1 has shown a prevalence of 70–75% [27,28]. 

However, it is important to consider that there are significant differences depending on the population analyzed, for example, 96.7% (Belgian subpopulation) [29] or 30.9% (Chinese subpopulation) [30,31]. As far as 17,27-M2 are concerned, the MB2 canal presents considerable variability, ranging between 10 and 95%. [27,29]. 

Considering the difference in the prevalence of MB2s (especially as regards the 17,27-M2) compared to the data present in the literature (the results of this study revealed the presence of MB2 in 65% of the 17,27-M2), it is possible to justify this finding by taking into consideration the fact that the incidence varies according to the method used in carrying out the scientific studies (sections of extracted teeth, SEM analysis, clearing of extracted teeth, micro-CT). It can therefore be deduced that the anatomy of the 17,27-M2 is extremely more complex than what can be highlighted by low-dose and wide FOV CBCT [30,31,32]. 

The “I” conformation was detected in rare cases, specifically in 3 16,26-M1 and in 4 upper 17,27-M2 (out of 52 16,26-M1 and 52 17,27-M2). 

Low-dose, wide FOV CBCT is not the exam of choice for this type of evaluation, but anatomy has been evaluated side by side with the prescription of this exam for other reasons, and this enhances the fact that the 3D investigation is fundamental and that can greatly simplify clinical practice [14,33].

Thus, it has been shown that MB2s have a small diameter in the 17,27-M2, and that consequently, they are often not detected in an examination of this type.

In the 17,27-M2, the presence of ribbon-like canals was found in greater numbers than in the 16,26-M1 (14 17,27-M2 altogether). It should be the objective of future studies to evaluate whether this could influence the choice of type of instrument, considering the chamber shape a prognostic factor of the difficulty of endodontic treatment. In this regard, future evaluations should be conducted to evaluate the relationship between the shape of the pulp chamber floor and the possible chamber accesses as a function of the endodontic treatment. Moreover, the number of MB2s detected at 5 mm from the CEJ is greater than that found in the apical 3 mm, a possible indication of the fact that the MB2 often flows into the mesial-vestibular or that it branches into an apical foramen independent of the mesial-vestibular, in which it is located in a more coronal position.

As a limitation of the present study, some measurements were performed inside the maxillary sinus; we believe that, although they may have modified the measurement when compared with the measurements inside the bone, they did not generate an error in the evaluation of the structures involved, also considering the small percentage. They certainly may have influenced the horizontal linear measurements near the apex but hardly the vertical ones. In addition to this evaluation, it is good to include within the limits the consideration in the present study of 17.27-M2 which presented nearby inclusions of wisdom teeth, which, with the enamel, could have influenced the horizontal measurement; although the canal was always clearly identifiable, they would be excluded from the inclusion criteria in case. It is evident that, considering the low dose of radiation administered to patients to obtain this radiographic investigation, the images 3 mm from the apex are less clear, whereas measurements lower than 0.5 mm become absolutely more complex.

This study was designed following a very simple intuitive classification able to fully understand all the examined teeth. It was therefore possible to simplify the type of anatomy, and possibly, it will be possible to project a type of endodontic access according to the radiographic conformation of the chamber floor, orienting the endodontic treatment from an operational point of view with an adequate choice of the most suitable rotary instruments.

## 5. Conclusions

The use of a diagnostic examination with a wide FOV and low radiation dose represents an exam capable of obtaining a lot of clinical information for endodontic treatment. Sometimes results can be superimposed on radiographic exams (micro-CT) or in vitro studies on extracted teeth. 

Regarding the schematic representation of the pulp chamber, by means of the classification proposed by Pawar and Singh, the 17,27-M2 show a simpler pattern than that frequent in the 16,26-M1. This study was designed following a very simple intuitive classification able to fully understand all the examined teeth and proven reliable for evaluating the positioning of the channels according to the schematic type of the pulp chamber, capable of orienting the type of opening to optimize the loss of healthy tissue or improve the stress to which the Ni-Ti rotary instruments are subjected.

Nevertheless, the endodontic anatomy of the 17,27-M2 represents a major challenge for the clinician due to its complexity both in canal shape and in ramification.

## Figures and Tables

**Table 1:** Descriptive characteristics of the evaluated parameters and linear measurements.

	16			17			26		27	
	Mean Value	SD	MeanValue		SD		Mean Value	SD	Mean Value	SD
Long diameter	5.89	1.01		5.74	0.98		5.98	1.05	5.62	0.99
Short diameter	3.74	1.51	3.25		1.42		3.18	1.62	3.2	
Shape	K(20), Y(4), I(2)			K(16), Y(9), I(1)			K(23), Y(2), I(1)		K(11), Y(12), I(3)	
At the orifice: M-B diameter	0.65	0.21		0.81	0.17		0.74	0.24	0.67	0.21
D-B diameter	0.69	0.11(2 NO)	0.72		0.14 (1 NO)		0.65	0.12 (1 NO)	0.69	0.15 (3 NO)
Palatal diameter	0.89	0.18		0.89	0.21		1.12	0.16	0.89	0.18
Mb2 diameter	0.38	0.12 (6 NO)		0.3	0.8 (10 NO)		0.39	0.9 (3 NO)	0.34	0.8 (15 NO)
At the orifice: MB-DB distance	2.33	0.22		2.35	0.21		2.22	0.2	2.19	0.2
DB-P distance	2.96	0.44		3.25	0.2		3.32	0.41	3.67	0.2
P-MB2 distance	3.65	0.52		3.39	0.42		3.62	0.48	3.68	0.39
MB1-MB2 distance	1.54	0.21		1.67	0.21		1.63	0.19	1.45	0.22
MB-P distance	4.91	0.3		5.03	0.36		5.02	0.26	4.87	0.31
5 mm from the CEJ: MB diameter	0.74	0.26		0.85	0.21		0.74	0.24	0.8	0.22
DB diameter	0.79	0.14 (2 NO)		0.65	0.11 (1 NO)		0.75	0.12 (1 NO)	0.68	0.12 (3 NO)
Palatal diameter	1.19	0.07		1.1	0.06		1.35	0.06	1.19	0.06
MB2 diameter	0.42	0.6 (2 NO)		0.47	0.5 (7 NO)		0.47	0.7 (3 NO)	0.42	0.4 (11 NO)
5 mm from the CEJ: MB-DB distance	4.35	0.82	2.88		0.62	4.13		0.78	2.97	0.64
DB-P distance	5.65	1.03		5.42	1.12	5.44		1.23	5.1	0.98
P-MB2 distance	5.94	1.45		4.94	1.44	5.61		1.65	3.28	1.12
MB1-MB2 distance	1.98	0.62		1.52	0.44	1.9		0.4	1.32	0.42
MB-P distance	7.56	1.7		6.55	1.55	7.14		1.52	5.68	1.49
5 mm from the CEJ: MB root diameter	6.78 2.64	1.76 0.68		6 2.8	1.35 0.70		6.46 2.75	2.02 1.12	5.96 2.8	1.68 0.56
DB root diameter	5.04 2.94	1.43 1.12 (3 NO)		4.49 3	1.06 0,89 (8 NO)		5.15 2.98	1.12 0.68	4.33 3.3	1.21 0.88 4 NO
Palatal root diameter	5.62 4.63	1.12 0.58		5.25 4.45	1.06 0.89	5.7	4.53	1.12 0.98	4.99 4.41	1.12 0.86
Roots fusions	8				19	7			18	
3 mm from the apex: MB diameter	0.54	0.22	0.69		0.18		0.66	0.12	0.55	0.11
DB diameter	0.54	0.21 (2 NO)	0.54		0.17 (1 NO)		0.58	0.18 (1 NO)	0.53	0.16 (3 NO)
Palatal diameter	0.87	0.12		0.86	0.12		0.82	0.16	0.59	0.17
MB2 diameter	0.39	0.08 (8 NO)		0.28	0.06 (9 NO)		0.44	0.12 (5 NO)	0.19	0.07 (7 NO)
3 mm from the apex: MB-DB distance	4.93	1.89		2.82	0.67		4.07	1.76	3.06	1.99
DB-P distance	8.6	2		7.12	1.9		8.12	2.6	6.64	1.89
P-MB2 distance	8.48	2.42		5.7	1.88		7.6	2.43 (12 NO)	5.55	1.68
MB1-MB2 distance	1.48	0.41		0.87	0.22		1.72	0.41	1.76	0.45
MB-P distance	9.13	2.12		7.23	1.99		8.85	2.43	6.48	1.88
3 mm from the apex: MB root diameter	5.03 2.6	1.23 0.86 (3 NO)		4.26 2.14	1.18 0.64 (1 NO)	2.7 4.9		1.23 0.58 (2 NO)	4.6 2.94	1.55 0.66 (7 NO)
DB root diameter	4.08 2.91	1.21 0.68 (5 NO)		3.5 2.44	0.9 0.66 (10 NO)		3.65 2.4	1.44 0.54 (5 NO)	3.58 2.58	1.23 0.66 (9 NO)
Palatal root diameter	4.9 3.7	1.77 1.01	3.38 1.95		1.12 0.66		4.8 3.5	1.23 0.88	4 3.15	1.12 0.99 (2 NO)

(M = mesial; B = Buccal; D = Distal; P = Palatal; CEJ = Cement–Enamel Junction).

## Data Availability

Complete study data can be requested from Dr. Rodolfo Reda or Dr. Valentina Bellanova.
